# Supplementary material for: Bacterial Contamination of Equine Dentistry Equipment—Effect of Cleaning and Disinfection
Source: Animals (Basel). 2021 Aug 5;11(8):2320. doi: 10.3390/ani11082320 (PMC8388488; doi:10.3390/ani11082320)
Supplement: Supplementary file 1 [file animals-11-02320-s001.zip › Supplement/Table S1 Bacterial load pilot study.pdf]

Table S1. Bacterial load on different parts of handpieces after dental procedures, after cleaning or disinfection and on handpieces taken from the cupboard in the dental care room. Colony forming units (CFU) is given in total CFU/surface sampled on handpieces. Other surfaces in the dental care room was sampled when the room was empty. Colony forming units (CFU) are given in CFU/cm².

**Sampling surface**	**Time of sampling**	**Cleaning substance**	**Disinfection substance**	**total CFU**	**CFU/cm²**
Straight LSH, external surface	NA[1], handpiece taken from cupboard in the dental care room	U[2], but probably clinic's routine = wipe with damp wiping paper	U, but probably clinic's routine = IET	223	NA
Angled LSH 1, external surface	NA, handpiece taken from cupboard in the dental care room	U, but probably clinic's routine = wipe with damp wiping paper	U, but probably clinic's routine = IET	420	NA
Angled LSH 2, external surface	NA, handpiece taken from cupboard in the dental care room	U, but probably clinic's routine = wipe with damp wiping paper	U, but probably clinic's routine = IET	520	NA
Straight LSH, inside of the coupling	NA, handpiece taken from cupboard in the dental care room	U, but probably clinic's routine = wipe with damp wiping paper	U, but probably clinic's routine = IET	2	NA
Angled LSH 1, inside of the coupling	NA, handpiece taken from cupboard in the dental care room	U, but probably clinic's routine = wipe with damp wiping paper	U, but probably clinic's routine = IET	16	NA
Angled LSH 2, inside of the coupling	NA, handpiece taken from cupboard in the dental care room	U, but probably clinic's routine = wipe with damp wiping paper	U, but probably clinic's routine = IET	3	NA
Straight LSH, small holes in top of the head	NA, handpiece taken from cupboard in the dental care room	U, but probably clinic's routine = wipe with damp wiping paper	U, but probably clinic's routine = IET	1	NA
Burr surface	NA, handpiece taken from cupboard in the dental care room	U, but probably clinic's routine = wipe with damp wiping paper	U, but probably clinic's routine = IET	7	NA
Burr surface	After dental procedure	NA	NA	400	NA
SH, external surface	After dental procedure	NA	NA	1100	NA
SH, shaft	After dental procedure	NA	NA	100	NA
SH, connection area at the bottom of the head of SH where it connects with the sheath	After dental procedure	NA	NA	209	NA
SH, seal in the connection area at the bottom of the head of SH where it connects with the sheath	After dental procedure	NA	NA	43	NA
SH, inside of the coupling	After dental procedure	NA	NA	1	NA
SH, shaft	After decontamination and lubrication	None but wipe with damp wiping paper	IET[3]	27	NA
SH, internal surface of the head of SH	After decontamination and lubrication	None but wipe with damp wiping paper	IET	26	NA
SH, connection area at the bottom of the head of SH where it connects with the sheath	After decontamination and lubrication	None but wipe with damp wiping paper	IET	160	NA
SH, internal surface of the connection area in the top part of the sheath of the SH where it connects with the head	After decontamination and lubrication	None but wipe with damp wiping paper	IET	5	NA
SH, external surface sheath	After decontamination and lubrication	None but wipe with damp wiping paper	IET	224	NA

Time of sampling	Cleaning substance	Disinfection substance	total CFU	CFU/cm^2	
Head support	NA, sampling took place when the dental care room was empty	U	U	NA	TNTC[4]
Workbench	NA, sampling took place when the dental care room was empty	U	U	NA	0,12
Workbench adjacent to washing-up sink	NA, sampling took place when the dental care room was empty	U	U	NA	0,48
Washing-up sink adjacent to drain	NA, sampling took place when the dental care room was empty	U	U	NA	2,15
Metal trolley	NA, sampling took place when the dental care room was empty	U	U	NA	4,7
Negative control	NA	NA	NA	0	NA

[1.] Not applicable, [2.] Unknown, [3.] Isopropanol and ethanol with tensid, [4.] Too numerous to count
