# Supplementary material for: Bacterial Contamination of Equine Dentistry Equipment—Effect of Cleaning and Disinfection
Source: Animals (Basel). 2021 Aug 5;11(8):2320. doi: 10.3390/ani11082320 (PMC8388488; doi:10.3390/ani11082320)
Supplement: Supplementary file 1 [file animals-11-02320-s001.zip › Supplement/Table S2 sampling methods tested.pdf]

Table S2. Sampling methods tested for sampling of handpieces, during pilot and main study. The samples were taken with Swab Samplers, copper wires formed to brushes, or a steel wire and all samples were placed in Swab Sampler tubes with letheen broth. Swab Sampler tubes were taken to the laboratory where they were vortexed and 1 ml broth was drawn from the sampling tube and put onto a 3M Petrifilm™ aerobic count (AC) Plate. All samples were analysed for total aerobic colony count (ACC).

Study	Sampling method	Sampling surface	Usability	Representativity
Pilot	Copper wires formed to brush	Inside of the coupling LSH/SH/HSH	Difficult to sample	Most likely not representative samples due to difficulties sampling the surface with chosen sampling method. Copper is antibacterial which may influence samples.
Pilot	Copper wires formed to brush	SH, seal in the connection area at the bottom of the head of SH where it connects with the sheath	Difficult to sample	Most likely not representative samples due to difficulties sampling the surface with chosen sampling method. Copper is antibacterial which may influence samples.
Pilot	Copper wires formed to brush	SH, internal surface of the connection area in the top part of the sheath of the SH where it connects with the head	Difficult to sample	Most likely not representative samples due to difficulties sampling the surface with chosen sampling method. Copper is antibacterial which may influence samples.
Pilot	Steel wire	Small holes in the top of the head of LHS	Difficult to sample	Most likely not representative samples due to difficulties sampling the surface with chosen sampling method.
Pilot	Swab	SH, connection area at the bottom of the head of SH where it connects with the sheath	A bit difficult to sample	Not representative surface to sample for internal surfaces.
Pilot	Swab	SH, external surface sheath	Possible to sample but can be integreated in sampling of the external surface of handpieces	Samples are most likely representative.
Pilot	Swab	Burr surface	Difficult to sample	Most likely not representative samples due to difficulties sampling the surface with chosen sampling method.
Pilot and main	Swab of body	External surface HSH/LSH/SH	Possible to standardize sampling	Samples are most likely representative.
Pilot and main	Swab of shaft	Shaft SH	Possible to standardize sampling	Most likely samples are not representative for internal surfaces of SH since other parts of the shaft, e.g. the surgical gear are probably more contaminated and more difficult to decontaminate
Main	Swab of coupling	Coupling HSH	Possible to standardize sampling	Probably representative but the method is not optimal for the surface of the coupling
Main	Swab of tunnel	Coupling LSH	Possible to standardize sampling	Most likely not representative since sampling is only possible in a small part of the tunnel. The sample is not representative for internal surfaces of the LSH.
