# Supplementary figures and images for: Bacterial Contamination of Equine Dentistry Equipment—Effect of Cleaning and Disinfection
Source: Animals (Basel). 2021 Aug 5;11(8):2320. doi: 10.3390/ani11082320 (PMC8388488; doi:10.3390/ani11082320)

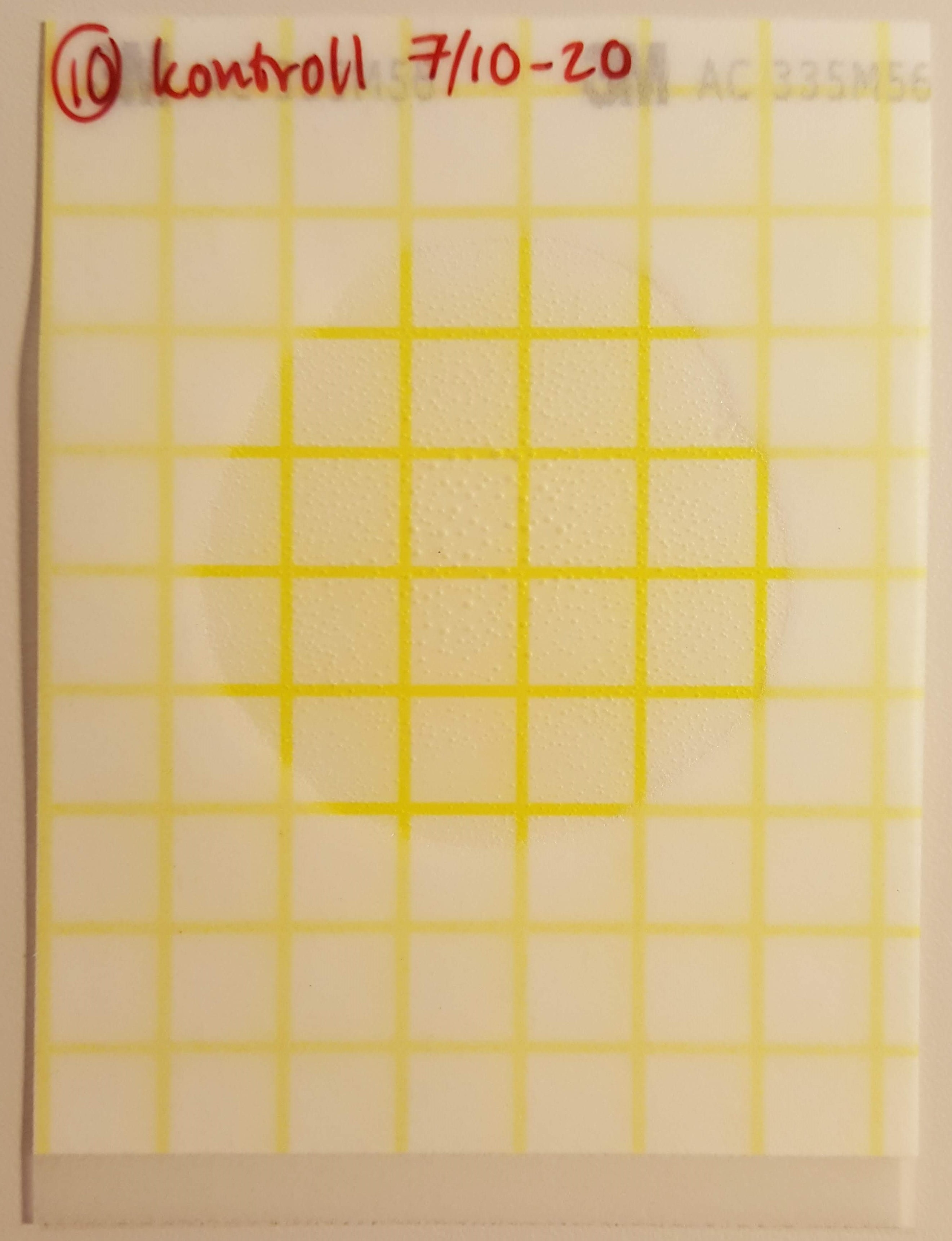

Supplement: Supplementary file 1 [file animals-11-02320-s001.zip › Supplement/Figure S1 PetrifilmTM negative control.jpg]
